# Isoprostanoids Levels in Cerebrospinal Fluid Do Not Reflect Alzheimer’s Disease

**DOI:** 10.3390/antiox9050407

**Published:** 2020-05-10

**Authors:** Carmen Peña-Bautista, Miguel Baquero, Marina López-Nogueroles, Máximo Vento, David Hervás, Consuelo Cháfer-Pericás

**Affiliations:** 1 Neonatal Research Unit, Health Research Institute La Fe, 46026 Valencia, Spain; carpebau93@gmail.com (C.P.-B.); maximo.vento@uv.es (M.V.); 2 Neurology Unit, University and Polytechnic Hospital La Fe, 46026 Valencia, Spain; miquelbaquero@gmail.com; 3 Analytical Unit Platform, Health Research Institute La Fe, 46026 Valencia, Spain; marina_lopez@iislafe.es; 4 Biostatistical Unit, Health Research Institute La Fe, 46026 Valencia, Spain; bioestadistica@iislafe.es

**Keywords:** Alzheimer’s disease, cerebrospinal fluid, biomarker, lipid peroxidation, mass spectrometry, blood-brain barrier

## Abstract

Previous studies showed a relationship between lipid oxidation biomarkers from plasma samples and Alzheimer’s Disease (AD), constituting a promising diagnostic tool. In this work we analyzed whether these plasma biomarkers could reflect specific brain oxidation in AD. In this work lipid peroxidation compounds were determined in plasma and cerebrospinal fluid (CSF) samples from AD and non-AD (including other neurological pathologies) participants, by means of an analytical method based on liquid chromatography coupled with mass spectrometry. Statistical analysis evaluated correlations between biological matrices. The results did not show satisfactory correlations between plasma and CSF samples for any of the studied lipid peroxidation biomarkers (isoprostanes, neuroprostanes, prostaglandines, dihomo-isoprostanes). However, some of the analytes showed correlations with specific CSF biomarkers for AD and with neuropsychological tests (Mini-Mental State Examination (MMSE), Repeatable Battery for the Assessment of Neuropsychological Status (RBANS)). In conclusion, lipid peroxidation biomarkers in CSF samples do not reflect their levels in plasma samples, and no significant differences were observed between participant groups. However, some of the analytes could be useful as cognitive decline biomarkers.

## 1. Introduction

Alzheimer’s Disease (AD) is the main cause of dementia worldwide and one of the most important causes of death in elderly people [1]. The demographic change in population highlights a possible increase in the impact of this pathology on society and the economy [2]. AD is characterized mainly by memory loss and progressive cognitive impairments that ends up incapacitating patients and prevent them from participating in the activities of daily life [3]. Histologically, AD is characterized by an intracellular and extracellular accumulation of phosphorylated tau (p-Tau) and β-amyloid proteins in neurons that leads to synapse loss [4]. However, the complete physiopathological mechanisms of the pathology are not completely understand.

Different metabolites from cerebrospinal fluid (CSF) samples have been studied as potential AD diagnosis biomarkers in order to improve the diagnosis accuracy of amyloid, total Tau (t-Tau) and p-Tau levels. In fact, α-synuclein determination complements actual AD biomarkers and allows to distinguish between mild cognitive impairment (MCI) due to AD and other MCI causes with better accuracy [5]. Furthermore, synaptosomal-associated protein-25 (SNAP-25), visinin-like protein 1 (VILIP-1), and chitinase3-like protein 1 (YKL-40) were altered at very early stages of the pathology [6], and cholecystokinin was related to a lower likelihood of MCI and better neuropsychological status [7]. However, CSF is obtained by means of a painful procedure that is not advisable for some individuals and it is not considered a population screening test. Nowadays, there is a growing field that is trying to find biomarkers in other biological fluids that could reflect brain damage from the disease [8]. Moreover, different authors found a correlation between biomarkers from CSF and plasma samples [9]. However, the correlation between these two biofluids for analytes (e.g., amyloid-β) is not clear and could depend on the analytical techniques used, as well as the effects of transportation through the brain-blood barrier (BBB) [10].

Neurodegenerative disorders show an important relationship with some oxidative stress mechanisms, especially lipid peroxidation [11]. In this sense, products originating from damage to lipid components of cellular membranes have been detected in the AD brain [12]. In addition, impaired lipid peroxidation levels have been found in peripheral fluids [13,14], offering an important step forward in non-invasive diagnosis [15]. Nevertheless, the interaction between the release of lipid peroxidation compounds by the central nervous system (CNS) and peripheral levels has not been evaluated.

The aim of this study was to measure a new set of lipid peroxidation products in CSF samples, and to evaluate their capacity to reflect neurodegeneration (correlation with amyloid and tau biomarker levels) and neuropsychological status (correlation with neuropsychological tests). In addition, we tried to establish a correlation between CSF and plasma lipid peroxidation biomarker levels in order to evaluate the latter as minimally invasive diagnosis biomarkers.

## 2. Materials and Methods

### 2.1. Study Design and Participants

The study protocol was approved by the Ethics Committee (CEIC) of the Health Research Institute La Fe (Valencia, Spain), (project reference number 2016/0257), and informed consent was obtained from all participants. Participants were recruited from the Neurology Unit at the University and Polytechnic Hospital La Fe (Valencia, Spain). Seventy-six patients aged between 50 and 75 years were included in the study. They were classified as AD (including mild cognitive impairment due to AD (MCI) and mild to moderate dementia due to AD) and non-AD (healthy controls and other dementias and cognitive impairments not caused by AD) groups. For this, they were subjected to neuropsychological tests (Repeatable Battery for the Assessment of Neuropsychological Status (RBANS), Clinical Dementia Rating (CDR), Mini-Mental State Examination (MMSE), Functional Activities Questionnaire (FAQ)) [16,17,18,19], structural neuroimaging by means of magnetic resonance imaging (MRI) or computerized axial tomography (CAT) [20], and CSF biomarkers (β-amyloid peptide (Aβ), t-Tau, p-Tau) [21,22]. The AD group was characterized by positive levels of Aβ, t-Tau and p-Tau and altered levels for neuropsychological evaluation by RBANS scale. In the non-AD group, participants with negative CSF biomarkers (Aβ, t-Tau and p-Tau) were included.

### 2.2. Samples Analysis

CSF samples (*n* = 76) were obtained by lumbar puncture as part of the diagnostic protocol in the Polytechnic University Hospital La Fe (Valencia, Spain), and they were kept at –80 °C. The analysis consisted of samples thawing on ice and adding 5 μL of the internal standard solution (IS) (d_4_-10-*epi*-10-F_4t_-NeuroP at 6 μmol L^−1^, and PGF2α-d_4_ at 10 μmol L^−1^) to 600 µL CSF, and they were diluted with 1300 μL of water. Then, a cleaning and pre-concentration step was carried out by solid-phase extraction (SPE) as described previously [14,15]. Briefly, the cartridges were conditioned (1 mL methanol and 1 mL H_2_O), then the samples were loaded, after that cartridges were washed (1 mL ammonium acetate (100 mmol L^−1^, pH 7) and 1 mL heptane). Finally, analytes were eluted (2 × 500 μL of methanol (5% *v/v* CH_3_COOH)). Then, samples were evaporated to dryness (vacuum evaporator) and reconstituted (100 μL of H_2_O (pH 3):CH_3_OH (85:15 *v/v*) with 0.01% (*v/v*) CH_3_COOH) to be injected into ultra-performance liquid chromatography coupled to tandem mass spectrometry (UPLC-MS/MS) (Waters Acquity UPLC-Xevo TQD system (Milford, MA, USA)).

Plasma samples from the same participants were analyzed by the method previously described by Peña-Bautista et al. [15].

### 2.3. Chromatographic System

The chromatographic system used consists of a Waters Acquity UPLC system coupled to a Xevo TQD system mass spectrometry system (Waters, United Kingdom). The HPLC conditions used were described in previous works [14,15].

### 2.4. Statistical Analysis

Differences between groups for numerical variables were analyzed by the Mann-Whitney test using SPSS version 20.0 software (SPSS, Inc., Chicago, IL, USA) and the values were expressed as median and interquartile range (IQR). Categorical variables were analyzed by the chi-square test. Finally, correlations among the biomarkers, as well as between the biofluids were analyzed by Pearson Correlation.

## 3. Results

### 3.1. Participants’ Characteristics

The clinical and demographic characteristics of the population are summarized in Table 1. There were no differences between groups for age and gender. By contrast, CSF biomarkers (Aβ, t-Tau and p-Tau) showed statistically significant differences between participant groups as was expected. The CSF Aβ levels were lower in the AD than in the non-AD patients. It could be explained by the aggregation of Aβ in the brain, hindering its transport to the CSF [23]. Similarly, the neuropsychological status (RBANS, MMSE, FAQ) showed differences between the groups while CDR did not show differences.

### 3.2. Correlation between CSF Isoprostanoids and Standard CSF Biomarkers

We analyzed possible correlations between the different isoprostanoids families (isoprostanes, neuroprotanes, dihomo-isoprostanes) (Figure S1), and CSF AD-specific biomarkers (Aβ, t-Tau, p-Tau) in order to establish a possible relationship between oxidative stress (brain grey and white matter damage) and amyloid pathology. Table 2 shows that Aβ correlates negatively with 7(*RS*)-ST-Δ^8^-11-dihomo-IsoF, 5-F_2t_-IsoP, total neurofurans and isofurans. In addition, p-Tau showed negative correlation with PGE_2_.

### 3.3. Correlations between CSF Isoprostanoids and Neuropsychological Evaluation

Regarding correlations between the isoprostanoids biomarkers and neuropsychological evaluation of the participants, Table 2 shows that RBANS and especially its visuospatial/constructional domain showed correlations with 15-F_2t_-IsoP, Ent-7(*RS*)-F_2t_-dihomo-IsoP and 15-keto-15-F_2t_-IsoP. The latter also showed correlation with the RBANS attention domain and with MMSE. Moreover, 15-keto-15-E_2t_-IsoP correlated with FAQ and CDR scores.

### 3.4. CSF and Plasma Lipid Peroxidation Biomarkers

A previous study described a diagnosis model for early AD based on the quantification of these isoprostanoid compounds in plasma samples. In the present study it was evaluated if these plasma levels reflected brain damage by means of the determination of the corresponding levels in CSF samples. In this sense, only 17(*RS*)-10-epi-SC-Δ^15^-11-dihomo-IsoF showed correlation between both matrices (PCC = 0.248, *p* = 0.031). In addition, when we analysed the results separately for AD and non-AD groups, we found that the non-AD group showed correlations between the two matrices for 15(*R*)-15-F_2t_-IsoP (PCC = 0.388, *p* = 0.024), 15-keto-15-F_2t_-IsoP (PCC = 0.360, *p* = 0.037) and 5-F_2t_-IsoP (PCC = 0.345, *p* = 0.046). However, these analytes did not show correlation between plasma and CSF samples in AD patients. In this AD group, 17-F_2t_-dihomo-IsoP (PCC = 0.399, *p* = 0.009) and 17(*RS*)-10-epi-SC-Δ^15^-11-dihomo-IsoF (PCC = 0.345, *p* = 0.045) showed correlation between CSF and plasma samples.

Table 3 shows the plasma levels of isoprostanoids biomarkers. Some metabolites showed statistically significant differences between the groups for 15(*R*)-15-F_2t_-IsoP (*p* < 0.001), 2,3-dinor-15-epi-15-F_2t_-IsoP (*p* = 0.028), 5-F_2t_-IsoP (*p* = 0.021), 15-F_2t_-IsoP (*p* < 0.001), PGF_2α_ (*p* = 0.011), neuroprostanes (*p* = 0.029), 10-epi-10-F_4t_-NeuroP (*p* < 0.001), isoprostanes (*p* < 0.001), Ent-7(*RS*)-7-F_2t_-dihomo-IsoP (*p* < 0.001), and 17-epi-17-F_2t_-dihomo-IsoP (*p* < 0.001). However, none of the CSF compounds showed statistically significant differences between the AD and non-AD groups.

## 4. Discussion

The reliable determination of lipid peroxidation product levels in CSF samples from biologically defined groups (AD and non-AD), based on specific AD biomarkers, was carried out. A previous study showed that these biomarkers were useful to diagnose AD with high accuracy when they were measured in plasma samples [15]. Previous studies also showed an increase of CSF isoprostanes in AD patients when their levels were corrected by ventricular volume, and these levels correlated with other clinical variables [24]; although Dutis et al. did not find any differences for CSF isoprostanes between AD, MCI and healthy control groups [25]. Therefore, ventricular volume could affect the concentration measured in CSF samples and that could be the reason why no differences were found between participant groups with or without AD.

In the present work, although isoprostanoids did not show differences between AD and non-AD groups, some lipid peroxidation products determined in CSF correlated with CSF Aβ and p-Tau levels. These results are consistent with those obtained by Kuo et al. who did not find differences between AD and non-AD groups for CSF levels of F2-isoprostanes and F4-neuroprostanes, but showed correlations with these metabolites and CSF Aβ levels [26]. By contrast, Yao et al. found that 12(S)-hydroxyeicosatetraenoic (HETE) acid and 15(S)-HETE correlated with CSF tau but not with CSF β-amyloid [27]. As amyloid biomarkers are specific for AD, isoprostanes seem to be more specific for amyloid pathology and AD than other biomarkers, such as HETE.

In our study, there is a correlation between isoprostanoids, such as 15-keto-15-F_2t_-IsoP, and cognitive impairments identified through MMSE scale examination. Similar results were obtained by Duits et al. that found a correlation between MMSE and F_2_-isoprostanes in ApoE Ɛ4 carriers [25]. Moreover, Kester et al. did not find differences for CSF isoprostanes levels between non-demented, MCI and AD patients, but these analytes showed an increase in the follow up of these participants showing an association with cognitive decline and MMSE examination [28]. In fact, CSF isoprostanes were described by de Leon et al. as good, not only in diagnosis, but also in AD progression study [29]. However, Yao et al. did not find any correlation between MMSE score and 12(S)-HETE and 15(S)-HETE, while in the present study 8-iso-15-keto-PGF_2α_ correlated with this neuropsychological status evaluation [27]. Therefore, ApoE Ɛ4 could be another important variable that affects isoprostanes levels in CSF.

In this study, correlations between lipid peroxidation levels in CSF and plasma samples were not found. Similarly, plasma and CSF levels of other metabolites, such as neurogranin, did not show any correlation [30]. Moreover, Aβ42 measured in plasma and CSF samples did not show any correlation [31], while Mehta et al. did not find correlation for Aβ40 and Aβ42 between these two biofluids [32]. However, Sun et al. studied correlations between different analytes such as α(1)-antichymotrypsin (ACT), α(1)-antitrypsin (AAT), interleukin-6 (IL-6), monocyte chemoattractant protein-1 (MCP-1) and oxidised low-density lipoprotein (oxLDL) between plasma and CSF samples. They found correlations for ACT, IL-6, MCP-1 and oxLDL, the latter showing a weaker correlation [33]. In addition, other analytes, such as adiponectin showed a correlation between these two matrices [34]. Moreover, different metabolites from the kyneurine pathway showed correlation between plasma and CSF samples, some showing a relationship with other CSF biomarkers (t-Tau, p-Tau) [9]. Therefore, metabolites exchange between BBB is not always equal, and concentrations between both biofluids could show differential distribution depending on the metabolite characteristics. As a hypothesis, CSF is continuously filtrating, so isoprotanes are not accumulated in this fluid, and the analyte concentrations in CSF are dependent on ventricular volume. By contrast, metabolites accumulating in the blood system for longer could be more easily measured. Previous studies showed that BBB permeability is increased under pathologic conditions, such as AD [35,36], and this permeability depends on inflammatory processes [37]. BBB alteration in AD could be responsible for the differences in correlation between plasma and CSF levels of different analytes in AD and non-AD. In addition, ventricular volume could influence the concentration of different metabolites in CSF, so corrections to this volume could result in a better correlation between plasma and CSF levels.

## 5. Conclusions

New lipid peroxidation biomarkers were satisfactorily measured in CSF samples from participants with AD and without AD (including healthy controls and other neurological pathologies) by an analytical method based on HPLC-MS/MS. These CSF metabolites are not able to discriminate between AD and non-AD groups, although some of them correlate with neuropsychological evaluations, as well as standard AD CSF biomarkers (β-amyloid, p-Tau). On the other hand, the levels of each isoprostanoid in plasma and CSF did not show correlation. It could be that changes in the transportation of substances through the BBB, the clearance of these compounds did not allow their accumulation and quantification in CSF, due to the necessity to correct CSF biomarker levels with ventricular volume. However, the CSF isoprostanoids levels could be useful in the evaluation of cognitive capacity.

## Figures and Tables

**Table 1:** Demographic and clinical variables of the study participants.

Variables	Non-AD (*n* = 34) ^$^	AD (*n* = 42)	*p*-Value (Mann-Whitney)
Age (years) Median (IQR)	66 (63, 72)	70 (68, 73)	0.102
Gender (Female) (*n*, %))	17 (50%)	28 (67%)	0.142
CSF β-amyloid (pg mL^−1^) Median (IQR)	1236.50 (950, 1435)	630 (535, 735)	0.000 *
CSF t-Tau (pg mL^−1^) Median (IQR)	230 (159, 347)	573 (436, 1005)	0.000 *
CSF p-Tau (pg mL^−1^) Median (IQR)	47 (32, 61)	86 (71, 122)	0.000 *
CDR Median (IQR)	0.5 (0, 0.5)	0.5 (0.5, 1)	0.071
MMSE Median (IQR)	27 (21, 28)	24 (18, 25)	0.004 *
RBANS.IM Median (IQR)	73 (69, 90)	57 (40, 67)	0.000 *
RBANS.V/C Median (IQR)	87 (75, 100)	75 (57, 87)	0.016 *
RBANS.L Median (IQR)	85 (60, 92)	60 (51, 82)	0.031 *
RBANS.A Median (IQR)	79 (60, 88)	60 (49, 79)	0.004 *
RBANS.DM Median (IQR)	68 (56, 88)	40 (40, 53)	0.000 *
FAQ Median (IQR)	3 (0, 8)	7 (3, 13)	0.015 *

* *p* < 0.05; IQR: inter-quartile range; RBANS.IM: Repeatable Battery for the Assessment of Neuropsychological Status–Immediate Memory; RBANS.V/C: RBANS–Visuospatial/Constructional; RBANS.L: RBANS–Language; RBANS.A: RBANS–Attention; RBANS.DM: RBANS–Delayed Memory; CDR: Clinical Dementia Rating values; FAQ: Functional Activities Questionnaire; CSF: cerebrospinal fluid. ^$^ The non-AD group is composed of healthy controls (*n* = 4) and other dementias and cognitive impairments not caused by AD (*n* = 30).

**Table 2:** Correlations between CSF isoprostanoids and clinical variables (standard CSF biomarkers, neuropsychological evaluation).

Correlations		CSF Aβ	CSF t-Tau	CSF p-Tau	CDR	MMSE	RBANS.IM	RBANS.V/C	RBANS.L	RBANS.A	RBANS.DM	FAQ
15*(R)*-15-F_2t_-IsoP	PCC	−0.196	−0.094	−0.032	−0.038	0.159	−0.030	0.124	−0.031	0.147	−0.022	−0.076
	*p*-value	0.089	0.419	0.783	0.770	0.226	0.818	0.344	0.811	0.262	0.865	0.564
PGE_2_	PCC	0.013	−0.205	−0.267	−0.031	0.095	0.136	0.043	0.219	0.106	0.061	−0.044
	*p*-value	0.908	0.076	0.020 *	0.814	0.471	0.298	0.743	0.092	0.418	0.643	0.738
2.3-dinor-15-epi-15-F_2t_-IsoP	PCC	−0.107	0.128	0.100	−0.047	0.081	0.010	0.021	0.019	0.074	−0.122	−0.025
	*p*-value	0.358	0.272	0.391	0.724	0.538	0.939	0.875	0.887	0.574	0.352	0.852
15-keto-15-E_2t_-IsoP	PCC	−0.088	−0.074	−0.015	0.297	−0.181	−0.113	−0.034	−0.037	−0.101	−0.120	0.275
	*p*-value	0.449	0.524	0.897	0.021 *	0.167	0.391	0.799	0.782	0.442	0.361	0.034 *
15-keto-15-F_2t_-IsoP	PCC	−0.109	−0.107	−0.101	−0.117	0.259	0.149	0.344	0.216	0.280	0.019	−0.230
	*p*-value	0.350	0.359	0.385	0.374	0.045 *	0.254	0.007 *	0.097	0.030 *	0.884	0.077
15-E_2t_-IsoP	PCC	−0.106	0.039	0.108	−0.137	0.072	0.086	−0.017	−0.047	0.146	0.051	−0.085
	*p*-value	0.360	0.741	0.353	0.296	0.587	0.514	0.895	0.724	0.265	0.697	0.517
5-F_2t_-IsoP	PCC	−0.242	−0.031	0.020	−0.005	0.103	−0.175	−0.079	−0.101	−0.032	−0.067	−0.050
	*p*-value	0.035 *	0.789	0.866	0.967	0.435	0.181	0.550	0.444	0.808	0.613	0.703
15-F_2t_-IsoP	PCC	−0.014	−0.068	−0.024	0.038	0.120	−0.022	0.265	−0.051	0.178	−0.007	−0.058
	*p*-value	0.903	0.562	0.834	0.773	0.360	0.870	0.041 *	0.699	0.173	0.959	0.659
PGF_2α_	PCC	−0.171	0.022	0.051	−0.075	0.031	−0.113	−0.138	−0.066	−0.070	−0.127	−0.097
	*p*-value	0.140	0.849	0.660	0.569	0.814	0.390	0.292	0.615	0.593	0.332	0.459
4*(RS)*-F_4t_-NeuroP	PCC	−0.018	−0.167	−0.130	−0.175	−0.049	0.109	−0.078	0.082	−0.123	−0.060	−0.149
	*p*-value	0.877	0.150	0.263	0.181	0.709	0.406	0.554	0.532	0.348	0.647	0.256
10-epi-10-F_4t_-NeuroP	PCC	−0.106	−0.045	−0.017	0.103	0.048	−0.077	0.068	−0.108	0.098	−0.047	0.015
	*p*-value	0.361	0.699	0.885	0.434	0.717	0.557	0.606	0.412	0.455	0.720	0.912
14*(RS)*-14-F_4t_-NeuroP	PCC	0.017	−0.167	−0.124	−0.074	0.071	0.029	−0.006	−0.006	0.135	0.105	−0.150
	*p*-value	0.886	0.150	0.284	0.574	0.591	0.824	0.965	0.962	0.304	0.423	0.252
Ent-7*(RS)*-7-F_2t_-dihomo-IsoP	PCC	−0.004	−0.081	−0.086	−0.050	0.186	0.055	0.349	0.011	0.240	−0.066	−0.173
	*p*-value	0.974	0.487	0.462	0.707	0.156	0.679	0.006 *	0.931	0.065	0.618	0.186
17-F_2t_-dihomo-IsoP	PCC	0.010	−0.086	−0.036	−0.009	−0.026	−0.099	0.153	−0.139	0.017	−0.102	−0.053
	*p*-value	0.935	0.460	0.760	0.947	0.842	0.451	0.242	0.290	0.899	0.440	0.688
17-epi-17-F_2t_-dihomo-IsoP	PCC	−0.003	−0.079	−0.073	−0.006	0.012	−0.129	0.076	−0.180	0.034	−0.014	−0.018
	*p*-value	0.982	0.497	0.530	0.963	0.928	0.326	0.564	0.168	0.797	0.914	0.893
17*(RS)*-10-epi-SC-Δ^15^-11-dihomo-IsoF	PCC	−0.093	0.014	−0.012	−0.055	0.226	0.026	0.170	−0.054	0.242	0.156	−0.014
	*p*-value	0.422	0.901	0.916	0.675	0.083	0.847	0.194	0.683	0.062	0.233	0.913
7*(RS)*-ST-Δ^8^-11-dihomo-IsoF	PCC	−0.262	0.030	0.035	0.048	−0.030	−0.155	−0.040	−0.230	−0.110	−0.029	0.131
	*p*-value	0.022 *	0.797	0.765	0.715	0.821	0.238	0.761	0.077	0.405	0.828	0.318
Isoprostanes ^$^	PCC	−0.196	−0.022	−0.020	−0.085	0.004	−0.150	−0.238	−0.193	−0.141	−0.040	0.010
	*p*-value	0.089	0.852	0.863	0.520	0.976	0.253	0.067	0.139	0.284	0.761	0.940
Neurorostanes ^$^	PCC	−0.001	−0.011	−0.033	0.102	−0.019	−0.077	0.207	−0.029	0.055	0.028	−0.026
	*p*-value	0.995	0.924	0.775	0.437	0.883	0.556	0.113	0.825	0.678	0.831	0.841
Neurofurans ^$^	PCC	−0.246	−0.032	0.019	−0.159	0.142	0.122	−0.008	−0.013	0.093	0.135	−0.057
	*p*-value	0.032 *	0.784	0.871	0.224	0.278	0.355	0.953	0.920	0.481	0.304	0.667
Isofurans ^$^	PCC	−0.309	0.013	0.062	−0.098	−0.051	−0.120	−0.084	−0.083	−0.083	−0.132	0.040
	*p*-value	0.007 *	0.914	0.595	0.458	0.698	0.359	0.525	0.530	0.527	0.315	0.760

PCC: Pearson correlation coefficient; * *p* < 0.05; ^$^ Total parameters.

**Table 3:** Concentrations of lipid peroxidation biomarkers in plasma samples.

Concentration (nmol L^−1^)	Non-AD (*n* = 34)	AD (*n* = 42)	*p*-Value Mann-Whitney
15*(R)*-15-F_2t_-IsoP Median (IQR)	0.075 (0, 0.231)	0.300 (0.188, 0.394)	<0.001 *
PGE_2_ Median (IQR)	0.050 (0, 0.100)	0.038 (0, 0.125)	0.590
2,3-dinor-15-epi-15-F_2t_-IsoP Median (IQR)	0 (0, 0)	0 (0, 0.006)	0.028 *
15-keto-15-E_2t_-IsoP Median (IQR)	0.150 (0, 0.250)	0.163 (0, 0.325)	0.541
15-keto-15-F_2t_-IsoP Median (IQR)	0.113 (0.044, 0.181)	0.225 (0.069, 0.331)	0.065
15-E_2t_-IsoP Median (IQR)	0.200 (0.100, 0.325)	0.213 (0.019, 0.525)	0.900
5-F_2t_-IsoP Median (IQR)	0.263 (0.056, 0.831)	0.700 (0.350, 1.125)	0.021 *
15-F_2t_-IsoP Median (IQR)	0 (0, 0)	0.020 (0.009, 0.035)	<0.001 *
PGF_2α_ Median (IQR)	0.238 (0.044, 0.363)	0.413 (0.194, 0.706)	0.011 *
4*(RS)*-F_4t_-NeuroP Median (IQR)	0 (0, 1.475)	1.100 (0.763, 1.425)	0.119
1a,1b-dihomo-PGF_2α_ Median (IQR)	0 (0, 0)	0 (0, 0)	0.219
10-epi-10-F_4t_-NeuroP Median (IQR)	0.225 (0.175, 0.281)	0.079 (0.025, 0.175)	<0.001 *
14*(RS)*-14-F_4t_-NeuroP Median (IQR)	0.300 (0.019, 0.850)	0.563 (0.131, 1.044)	0.316
Ent-7*(RS)*-7-F_2t_-dihomo-IsoP Median (IQR)	0 (0, 0.050)	0.075 (0.050, 0.150)	<0.001 *
17-F_2t_-dihomo-IsoP Median (IQR)	0 (0, 0)	0 (0, 0)	0.096
17-epi-17-F_2t_-dihomo-IsoP Median (IQR)	0 (0, 0)	0 (0, 0.025)	<0.001 *
17*(RS)*-10-epi-SC-Δ^15^-11-dihomo-IsoF Median (IQR)	0 (0, 0)	0 (0, 0)	0.066
7*(RS)*-ST-Δ^8^-11-dihomo-IsoF Median (IQR)	0.013 (0, 0.050)	0.025 (0, 0.075)	0.098
Isoprostanes ^$^ Median (IQR)	0.449 (0.396, 0.488)	0.345 (0.234, 0.409)	<0.001 *
Neuroprostanes ^$^ Median (IQR)	0.142 (0.050, 0.207)	0 (0, 0.268)	0.029 *
Isofurans ^$^ Median (IQR)	0.073 (0.058, 0.105)	0.085 (0.069, 0.115)	0.202
Neurofurans ^$^ Median (IQR)	0.114 (0.082, 0.173)	0.095 (0, 0.169)	0.111

^$^ Arbitrary units: intensity of signal units x (internal standard concentration, mg L ^−1^); * *p*-value < 0.05.

## Supplementary Materials

The following are available online at https://www.mdpi.com/2076-3921/9/5/407/s1, Figure S1: Chemical structures of isoprostanes, dihomo-isoprostanes, and neuroprostanes.
