# Supplementary figures and images for: Isoprostanoids Levels in Cerebrospinal Fluid Do Not Reflect Alzheimer’s Disease
Source: Antioxidants (Basel). 2020 May 10;9(5):407. doi: 10.3390/antiox9050407 (PMC7278667; doi:10.3390/antiox9050407)

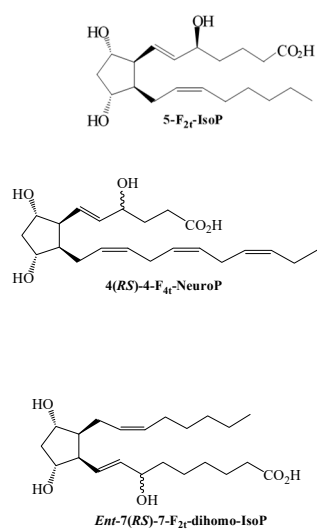

**Figure S1.** Chemical structures of isoprostanes, dihomo-isoprostanes, and neuroprostanes.

Supplement: Supplementary file 1 [file antioxidants-09-00407-s001.pdf]
